# Osteopontin facilitates tumor metastasis by regulating epithelial–mesenchymal plasticity

**DOI:** 10.1038/cddis.2016.422

**Published:** 2016-12-29

**Authors:** Rongjie Jia, Yingchao Liang, Rui Chen, Guoke Liu, Hao Wang, Min Tang, Xuyu Zhou, Huajing Wang, Yang Yang, Huafeng Wei, Bohua Li, Yipeng Song, Jian Zhao

**Affiliations:** 1 International Joint Cancer Institute, The Second Military Medical University, 800 Xiangyin Road, Library Building 9-11th Floor, Shanghai 200433, China; 2 The 305 Hospital of PLA, A13 Wenjin Street, Beijing 100017, China; 3 Department of Radiation Oncology, Yantai Yuhuangding Hospital, 20 East Yuhuangding Road, Yantai, Shandong 264000, China; 4 Changhai Hospital, The Second Military Medical University, 168 Changhai Road, Shanghai 200433, China; 5 PLA General Hospital Cancer Center Key Lab, PLA Postgraduate School of Medicine, 28 Fuxing Road, Beijing 100853, China

## Abstract

Tumor metastasis leads to high mortality; therefore, understanding the mechanisms that underlie tumor metastasis is crucial. Generally seen as a secretory protein, osteopontin (OPN) is involved in multifarious pathophysiological events. Here, we present a novel pro-metastatic role of OPN during metastatic colonization. Unlike secretory OPN (sOPN), which triggers the epithelial–mesenchymal transition (EMT) to initiate cancer metastasis, intracellular/nuclear OPN (iOPN) induces the mesenchymal–epithelial transition (MET) to facilitate the formation of metastases. Nuclear OPN is found to interact with HIF2*α* and impact the subsequent AKT1/miR-429/ZEB cascade. *In vivo* assays confirm that the progression of metastatic colonization is accompanied by the nuclear accumulation of OPN and the MET process. Furthermore, evidence of nuclear OPN in the lung metastases is exhibited in clinical specimens. Finally, VEGF in the microenvironment was shown to induce the translocation of OPN into the nucleus through a KDR/PLC*γ*/PKC-dependent pathway. Taken together, our results describe the pleiotropic roles of OPN in the tumor metastasis cascade, which indicate its potential as an effective target for both early and advanced tumors.

Metastasis is responsible for the majority of cancer-associated deaths. Plasticity of the cellular phenotype is critical for tumor metastasis. Epithelial–mesenchymal transition (EMT) endows epithelial-derived tumor cells with migratory and invasive properties, which facilitates the escape from primary sites in the early stage of metastatic dissemination.^1, 2^ Morphological analysis revealed similarities of metastatic lesions and primary tumors,^3, 4^ and miR-200s promote distant metastatic colonization by inducing mesenchymal–epithelial transition (MET).^5^ These findings suggest that MET, the reversal of EMT, can convert disseminated mesenchymal tumor cells back to an epithelial state in the later stage of metastatic colonization. Taken together, EMT and MET function in the initiation and termination stage of tumor metastasis, respectively. The crucial role of EMT has been well-recognized, but relatively few studies have focused on the role and mechanism of MET.

Considered to be a canonical secretory protein, the multifunction of osteopontin (OPN) has been extensively studied in the past.^6^ High cytoplasmic OPN staining was observed in multiple tumors and the OPN level is closely correlated with the pathological stage.^7^ Blocking OPN in breast cancer cells was reported to decrease the expression of SNAIL, SLUG, and TWIST, which suggests that secretory OPN (sOPN) is critical in EMT and tumor metastasis.^8^

However, knockout phenotypes cannot be fully rectified by treatment of the *OPN-*knockout mice or cells with recombinant OPN, and antibody neutralization of OPN in the wild-type mice or cells did not reproduce knockout phenotypes.^9^ In our previous work, recombinant human OPN (rhOPN) did not fully compensate for the weakened metastatic ability of HCC cells by OPN interference.^10^ Researchers gradually recognized that intracellular OPN (iOPN) existed and functioned separately from sOPN. Since the first introduction,^11^ three types of iOPN have been discovered: perimembranous,^12, 13^ nuclear,^14^ and cytoplastic.^15^ All these studies suggest that iOPN may have important roles in various cytological behaviors. However, the role of iOPN in cancer progression remains largely elusive.

Here, we investigated the pro-metastatic mechanism of OPN from a brand-new perspective. In accord with previous studies,^8, 16^ sOPN induced EMT in cancer; whereas the nuclear OPN triggered MET through the AKT1/miR-429/ZEB axis through interaction with HIF2*α*. *In vivo* studies and patient samples further supported the diverse roles of sOPN and iOPN in tumor metastasis.

## Results

### Expression of OPN in cancer cells

**Figure 1:**
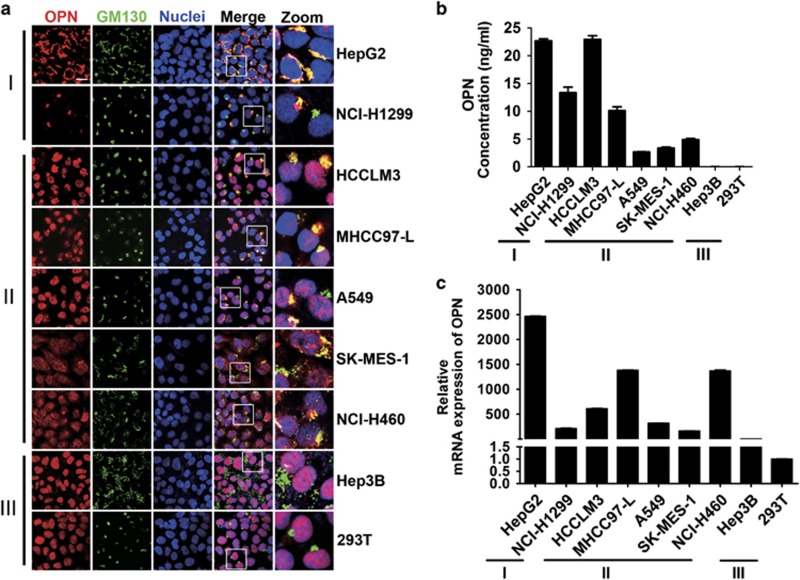
Expression of OPN in cancer cells. (**a**) Confocal microscopy of OPN and GM130 in liver cancer cell lines (HepG2, HCCLM3, MHCC97-L, and Hep3B), lung cancer cell lines (NCI-H1299, A549, SK-MES-1, and NCI-H460) and 293T. According to the expression of OPN, these cells are divided into different groups as denoted. Scale bar, 10 *μ*m. (**b**) Cell culture media were collected and secreted OPN was quantitated by ELISA. (**c**) Fold-change in mRNA level of OPN in indicated cells. Data represent mean±S.D.

Long regarded as a canonical secretory protein, OPN was recently identified to function intracellularly. The secretory form of OPN (sOPN), localized in the Golgi apparatus, contains an N-terminal signal sequence, which allows it to target secretory vesicles and be secreted extracellularly. To analyze the distinct expression of OPN in cancer cells, dual immunofluorescence staining of OPN and Golgi matrix protein 130 (GM130), the Golgi marker, was performed. Cancer cells can be classified into three types according to the OPN expression (Figure 1a). Type I cells contain only sOPN, such as HepG2 and NCI-H1299. Type II cells contain both secretory and intracellular/nuclear forms of OPN, such as HCCLM3, MHCC97-L, A549, SK-MES-1, and NCI-H460. Type III cells contain only intracellular/nuclear form of OPN (iOPN), such as Hep3B and 293T. Evident nuclear staining of OPN was also observed in the colon cancer cell lines and breast cancer cell lines (Supplementary Figure S1a).

The results of ELISA assays were consistent with the above data (Figure 1b; Supplementary Figure S1b). Secreted OPN was detected in the supernatant of type I and type II cells, but not in type III cells, even though OPN was expressed considerably (Figure 1c).

### sOPN triggers EMT whereas iOPN induces MET

**Figure 2:**
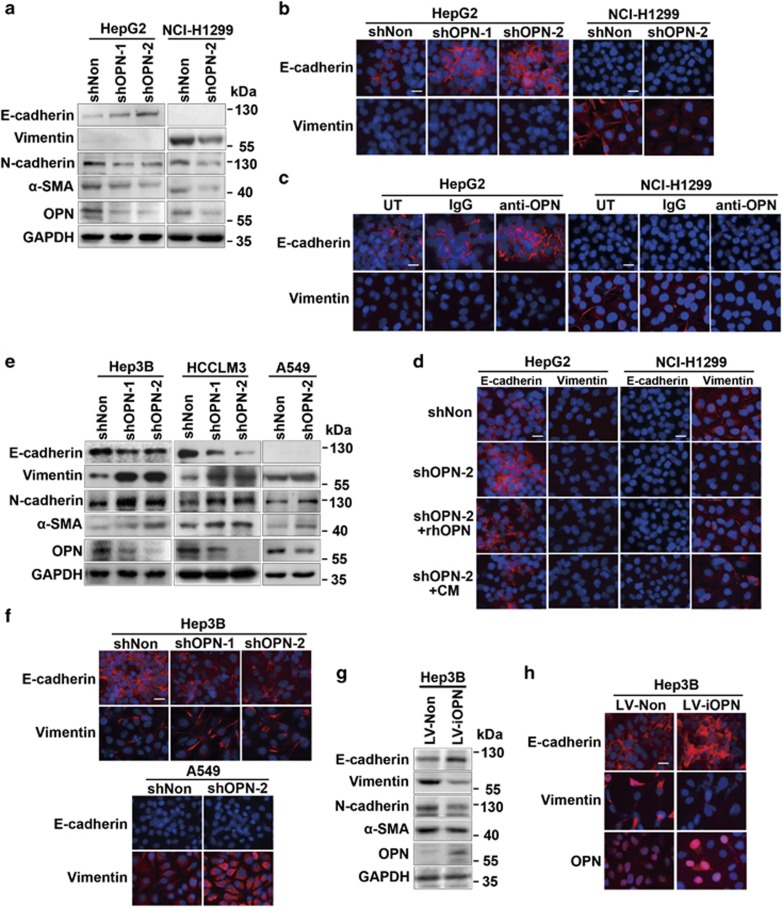
sOPN triggers EMT, whereas iOPN induces MET. (**a**, **e** and **g**) Immunoblot analysis of epithelial marker (E-cadherin) and mesenchymal markers (Vimentin, N-cadherin, and *α*-SMA) in HepG2, NCI-H1299, Hep3B, HCCLM3, and A549 cells transfected with the indicated lentivirus. (**b**, **f** and **h**) Immunofluorescence staining of E-cadherin and Vimentin in HepG2 and NCI-H1299 cells transfected with denoted lentivirus. Scale bars, 10 *μ*m. (**c**) Immunofluorescence staining of E-cadherin and Vimentin in HepG2 and NCI-H1299 cells, which were pretreated with control IgG or anti-OPN Ab (at 40 *μ*g/ml). (**d**) Staining of E-cadherin and Vimentin in HepG2 and NCI-H1299 cells, which were first transfected with the indicated lentivirus and then exposed to rhOPN (40 ng/ml) or conditioned medium (CM). Scale bars, 10 *μ*m

Consistent with previous reports, the blockage of sOPN by shOPN lentivirus in type I cells HepG2 and NCI-H1299 was able to trigger MET featured by the gain of epithelial markers, such as E-cadherin, and the loss of mesenchymal markers, such as N-cadherin, Vimentin, and *α*-SMA (Figure 2a). Levels of EMT-related transcription factors were greatly decreased (Supplementary Figure S2a). Immunofluorescence showed enhanced membranous staining of E-cadherin and reduced Vimentin staining in OPN-silenced or antibody-neutralized HepG2 or NCI-H1299 cells (Figures 2b and c). Furthermore, OPN silencing induced the gain of E-cadherin or loss of Vimentin, which was reversed when HepG2 or NCI-H1299 cells were exposed to the rhOPN or conditioned media (CM) (Figure 2d). Thus, sOPN was able to trigger EMT.

Interestingly, when OPN was down-regulated in type III and type II cells Hep3B, HCCLM3, and A549, E-cadherin was decreased, whereas N-cadherin, Vimentin, and *α*-SMA, as well as EMT-related transcription factors ZEB1 and ZEB2 were increased (Figure 2e; Supplementary Figure S2b). The reduced membranous staining of E-cadherin and increased Vimentin staining were observed in OPN-silenced Hep3B and A549 cells (Figure 2f). In addition, lentivirus encoding iOPN cDNA was employed to overexpress iOPN in Hep3B cells, which had relatively low level of OPN. The enforced expression of iOPN in Hep3B led to increase of E-cadherin and decrease of Vimentin, N-cadherin, and *α*-SMA, and the EMT-activating transcription factors decreased as well (Figure 2g; Supplementary Figure S2c). The restoration of E-cadherin and loss of Vimentin were also observed, and the overexpressed iOPN was located in the nucleus (Figure 2h). Thus, iOPN may induce MET via ZEB1 and ZEB2.

These results suggest that sOPN and iOPN have diverse effects on the EMT/MET process in cancer cells.

### miR-429 has a crucial role in iOPN-induced MET

**Figure 3:**
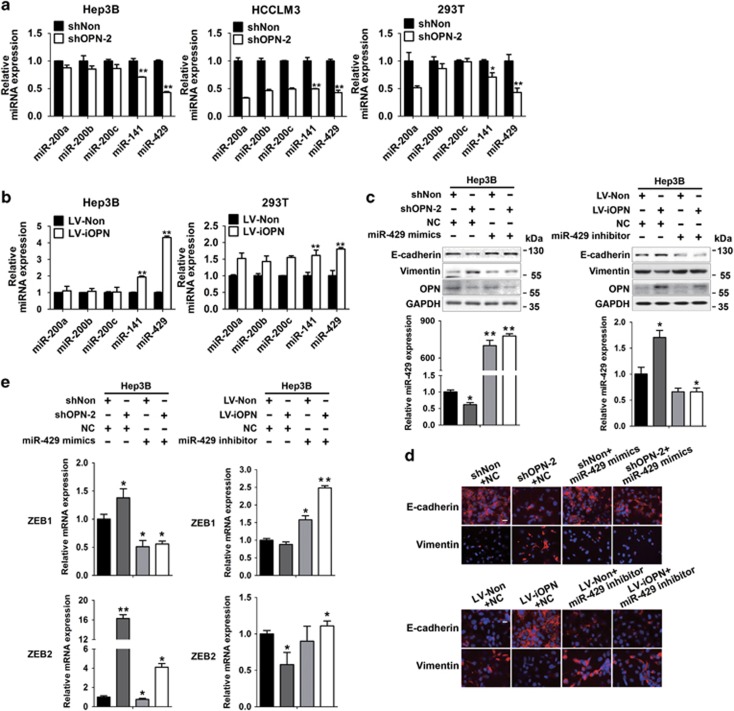
miR-429 has a crucial role in iOPN-induced MET. (**a** and **b**) Relative expression levels of miR-200 family in Hep3B, HCCLM3, and 293T cells transfected with denoted lentivirus. (**c** and **d**) Immunoblot and immunofluorescence analysis of E-cadherin and Vimentin in Hep3B cells simultaneously transfected with indicated lentivirus and miR-429 mimics or inhibitor. Scale bars, 10 *μ*m. (**e**) Fold-change in mRNA levels of EMT-related transcriptional factors ZEB1 and ZEB2 in Hep3B cells treated as represented. Data represent mean±S.D. *P<0.05, **P<0.01

Many studies have focused on sOPN and EMT regulation,^8, 17^ but little is known about iOPN and MET regulation. The microRNA-200 family (miR-200s), including miR-200a, miR-200b, miR-200c, miR-141, and miR-429, has been reported to promote MET by direct targeting the ZEB/E-cadherin axis.^18, 19^ Given that iOPN greatly regulates *ZEB1* and *ZEB2* expression (Supplementary Figure S2), the expression of miR-200s was examined in Hep3B, HCCLM3, and 293T cells. Alterations of miR-141 and miR-429 could be found in all the groups tested, when OPN was either silenced in Hep3B, HCCLM3, and 293T cells (Figure 3a) or overexpressed in Hep3B and 293T cells (Figure 3b). The upregulation of miR-141 and miR-429 was accompanied by the increase of iOPN and vice versa. miR-141/miR-429 mimics was further transfected with OPN knockdown, or miR-141/miR-429 inhibitor with nuclear OPN overexpression in Hep3B cells. miR-429 mimics was found to reverse the decrease of E-cadherin and increase of Vimentin caused by OPN deficiency, whereas miR-429 inhibitors reversed the increase of E-cadherin and decrease of Vimentin induced by nuclear OPN overexpression (Figures 3c and d). In addition, miR-429 mimics attenuated the increase of ZEB1 and ZEB2 expression induced by OPN deficiency, and miR-429 inhibitor reversed the decrease of ZEB1 and ZEB2 expression caused by enforced iOPN expression (Figure 3e). However, treatment with miR-141 mimics or inhibitor was unable to achieve the similar results (Supplementary Figure S3). Taken together, miR-429 has a crucial role in iOPN-induced MET.

### iOPN induces miR-429 expression by regulating AKT1 expression

**Figure 4:**
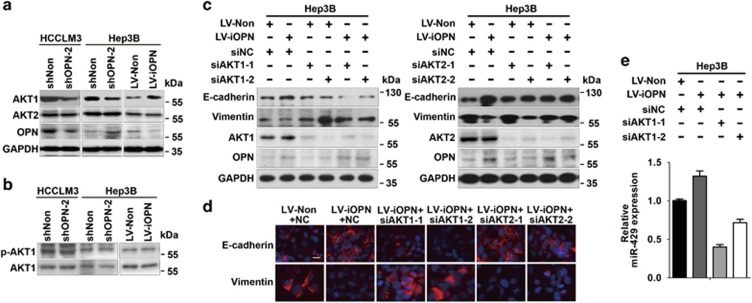
iOPN induces miR-429 expression by regulating AKT1 expression. (**a** and **b**) Immunoblot analysis of AKT1, AKT2 and p-AKT1 in HCCLM3 and Hep3B cells transfected with indicated lentivirus. (**c** and **d**) Immunoblot and immunofluorescence analysis of E-cadherin and Vimentin in Hep3B cells transfected with denoted lentivirus and siRNA targeting AKT1 or AKT2. Scale bar, 10 *μ*m. (**e**) Quantitative PCR analysis of miR-429 in Hep3B cells treated as indicated. Data represent mean±S.D.

The close relationship between OPN and AKT has been extensively investigated. Generally, OPN activates AKT by the phosphorylation at serine-473.^20, 21^ Moreover, AKT1 and AKT2 have been reported to have different roles in regulating miR-200s and EMT. Lower abundance of miR-200s was observed in *AKT1*-knockout mammary adenocarcinomas than in wild-type or *AKT2*-knockout ones,^22^ and downregulation of AKT1 rather than AKT2 in IGF-1R-stimulated cells could induce EMT.^23^ We therefore detected AKT1 and AKT2 expression with altered expression of OPN. The expression of AKT1 declined in OPN-deficient group or increased in nuclear OPN-enforced group (Figure 4a). However, alterations of the AKT2 level were less obvious. Moreover, the level of phosphorylated AKT1 barely changed (Figure 4b). Thus, different from sOPN, iOPN may regulate the expression level of AKT1 rather than its phosphorylation. Then, siAKT1 and siAKT2 were used in iOPN-overexpressed Hep3B cells to determine the role of AKT1 and AKT2 in iOPN-induced MET. Blocking AKT1 reversed the increase of E-cadherin and decrease of Vimentin evoked by nuclear OPN, whereas AKT2 blockage failed to achieve this effect (Figures 4c and d). Moreover, iOPN-induced upregulation of miR-429 was diminished when AKT1 was knocked down (Figure 4e). These data suggest that iOPN may upregulate AKT1 and miR-429 expression.

### iOPN regulates AKT1 expression by interacting with HIF2*α*

**Figure 5:**
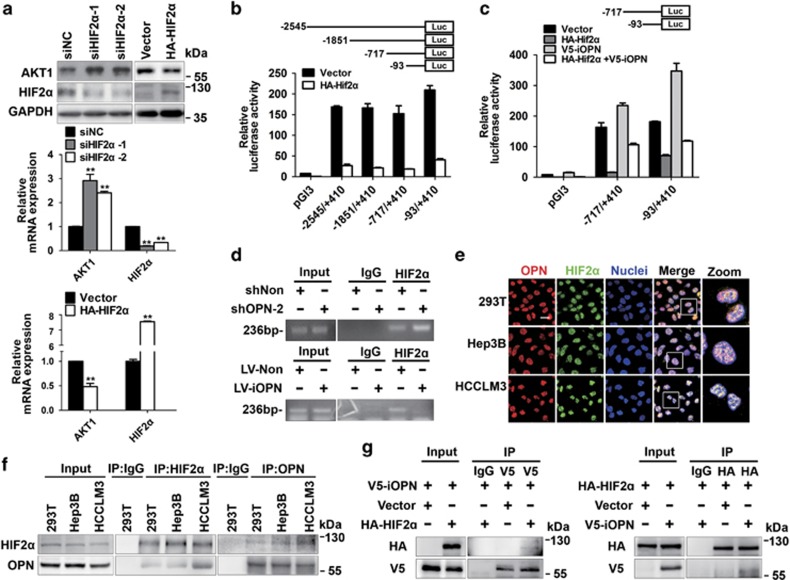
iOPN regulates AKT1 expression by interacting with HIF2*α*. (**a**) Immunoblot and qPCR analysis of AKT1 in Hep3B cells with knockdown or overexpression of HIF2*α*. *P<0.05, **P<0.01. (**b** and **c**) Dual luciferase reporter assay of *AKT1* promoter mutants in collected 293 T cells 48 h after indicated transfection. Each sample was performed in triplicate. Data represent mean±S.D. (**d**) In Hep3B cells transfected with the indicated lentivirus, chromatin was immunoprecipitated using indicated antibodies, corresponding DNA fragments were then amplified and measured. (**e**) Confocal microscopy of OPN and HIF2*α* in 293 T, Hep3B, and HCCLM3 cells. Images are representative of three experiments. Scale bar, 10 *μ*m. (**f**) lysates from 293 T, Hep3B, and HCCLM3 cells were immunoprecipitated with denoted antibodies. HIF2*α* and OPN levels were then assessed. (**g**) Co-immunoprecipitation (Co-IP) analysis of V5-iOPN and HA-HIF2*α* proteins

The mechanism by which iOPN regulates AKT1 expression was then explored. In the promoter region of *AKT1*, we found three hypoxia-responsive elements (HREs), GGGCGTGG, GTACGTGG, and AGGCGTGC, located at nt-1444, nt-482, and nt+306, respectively. In a study using the yeast two-hybrid screen analysis, OPN was shown to interact with various proteins, including EPAS1 (HIF2*α*).^24^ Accordingly, we boldly hypothesized that iOPN may combine with HIF2*α* and regulate *AKT1* transcription. Indeed, HIF2*α* was found to act as a negative regulator of *AKT1* at both protein and mRNA level (Figure 5a). To investigate how HIF2*α* affects *AKT1* expression, various truncated mutants of the *AKT1* promoter were constructed based on HREs. Transcriptional activities were measured in Hep3B cells, and HIF2*α* might target nt-93 to nt+410 cis-regulatory elements to repress *AKT1* transcription (Figure 5b). Moreover, ectopic iOPN expression could increase the activity of *AKT1* promoter and partially reverse HIF2*α*'s inhibition of *AKT1* transcription (Figure 5c). We next sought to determine whether the binding of HIF2*α* to *AKT1* promoter was regulated by iOPN. Specific primers of three HREs were used in chromatin immunoprecipitation (ChIP) assays and only the second fragment was successfully detected, and the binding of HIF2*α* to *AKT1* promoter was enhanced in OPN-deficient cells or decreased in iOPN-overexpressed cells (Figure 5d). Thus, iOPN negatively regulates the binding of HIF2α to *AKT1* promoter.

The interaction between iOPN and HIF2*α* was then investigated. Immunofluorescence staining showed co-localization of OPN and HIF2*α* in the nuclei of 293T, Hep3B and HCCLM3 cells (Figure 5e). In addition, endogenous iOPN and HIF2*α* could be co-immunoprecipitated in these cell lines (Figure 5f). Further Co-IP assays confirmed the interaction between nuclear OPN and HIF2*α* (Figure 5g).

Taken together, the interaction of iOPN with HIF2*α* in the nucleus could negatively regulate the binding of HIF2*α* to *AKT1* promoter and decrease the inhibitory effect of HIF2*α* on *AKT1* transcription, resulting in the upregulation of AKT1 and miR-429.

### OPN deficiency impairs MET and metastatic colonization in the distant organ

**Figure 6:**
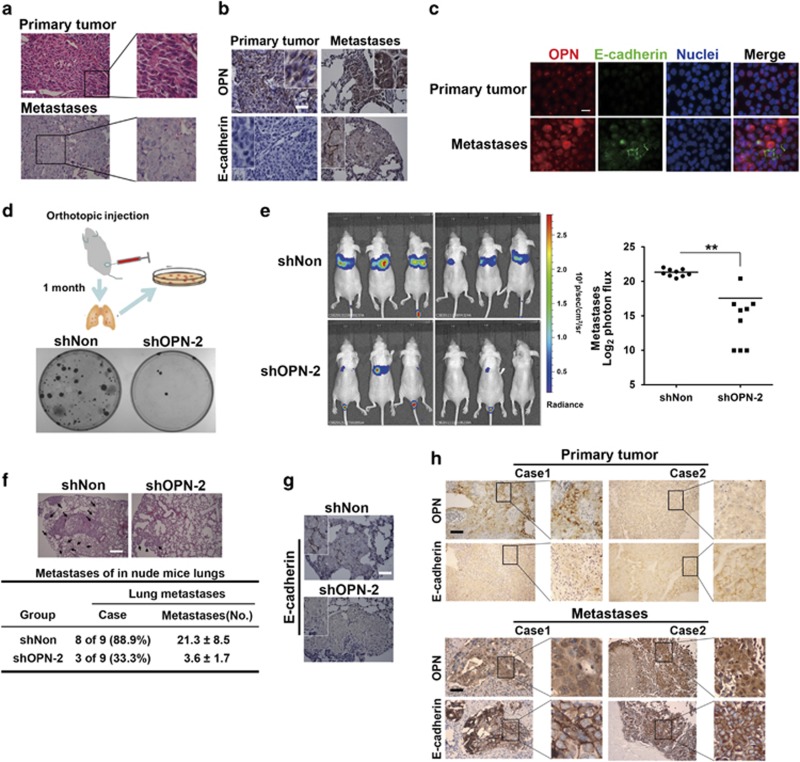
OPN deficiency impairs MET and metastatic colonization in the distant organ. (**a**) Representative images of H&E-stained primary tumor lesions and pulmonary metastases from animals subcutaneously injected with HCCLM3 cells. Scale bar, 40 *μ*m. (**b** and **c**) IHC and immunofluorescence staining of OPN and E-cadherin in primary tumors and matched lung metastases. Scale bars, 40 *μ*m (**b**) and 10 *μ*m (**c**). (**d**) Plate colony formation of HCCLM3 cells isolated from metastatic lungs. Schema is displayed. (**e**) Representative luminescent images and the log2 luminescent photon flux of mice 30 days after tail-vein injection of indicated HCCLM3 cells, which were labeled with luciferase. **P<0.01. (**f** and **g**) Representative images of hematoxylin and eosin (H&E) and IHC staining of lung sections from the mice after tail-vein injection. Dark arrowheads mark metastatic nodules and metastases in lungs were counted. Scale bar, 100 *μ*m. (**h**) Representative images of IHC staining in the primary tumors and metastases lesions from HCC patients. Case 1 and 2 of metastases are from the lumbar soft tissue and the cervical lymph node, respectively. Scale bars, 100 *μ*m

MET occurs in the late phase of distant metastasis. Several studies have demonstrated the restoration of epithelial properties of tumor cells in metastases.^3, 4^ Morphological analysis showed cells in the metastatic nidus were round, cuboidal and closely arranged, whereas in corresponding primary tumor foci, cells were spindle-shaped and mesenchymal-like (Figure 6a). Immunohistochemical (IHC) staining showed that E-cadherin, a pivotal hallmark of epithelial status, appeared in the membrane in lung metastases, whereas no obvious E-cadherin expression was detected in primary tumor cells; and OPN expression was increased in the nucleus of metastases (Figure 6b). In addition, cells acquired from resected primary and metastatic nodules were cultured *in vitro* and subjected to immunofluorescence staining. Consistent with IHC staining, the expressions of membranous E-cadherin and nuclear OPN were significantly higher in cells from lung metastases (Figure 6c). In addition, plate colony-forming assay showed the OPN-decreased group formed fewer tumor colonies, suggesting lung colonization relied on iOPN expression (Figure 6d). These results suggested the establishment of MET agreed with the dramatic increase of nuclear OPN during metastatic colonization in distant organs.

Moreover, miR-429 expression was higher in metastatic colonies or lung-metastatic HCCLM3 cells (Supplementary Figures S4a and b), which were both in keeping with above data. The overexpression of miR-200s promoted metastatic colonization partly through the direct inhibition of Sec23a expression, which reduces the secretion of metastasis-related proteins, including OPN.^5^ miR-429 was found to decrease the expression of Sec23a but not OPN (Supplementary Figure S4c). Mass spectrometry analysis and immunoblotting of CM from Sec23a-silenced HCCLM3 cells showed a global reduction of OPN secretion (Supplementary Figures S4d and e). And immunofluorescence staining showed that OPN was detained intracellularly due to the reduced secretion caused by Sec23a silencing (Supplementary Figure S4f). These data suggest that the nuclear accumulation of OPN in lung metastases resulted in the upregulation of miR-429 and the subsequent suppression of Sec23a expression and OPN secretion, which orchestrated the MET process induced by nuclear OPN.

To verify the impact of OPN on MET and metastasis, we constructed a metastatic model and detected lung metastases via the bioluminescence imaging system. The signal intensity of lung metastases in the shOPN group was remarkably weaker (Figure 6e). In addition, H&E and IHC staining showed that OPN deficiency led to fewer metastases and diminished membranous E-cadherin (Figures 6f and g). These results suggested that iOPN expression is related to MET, which contributes to metastatic colonization in the distant organ.

Furthermore, we analyzed OPN and E-cadherin expression in clinical specimens. In primary tumors of 158 HCC patients, strong cytoplasmic OPN staining and weak E-cadherin expression was observed in most cases (Figure 6h, case 1); in other cases, the low cytoplasmic expression of OPN was accompanied by the membranous location of E-cadherin (Figure 6h, case 2). OPN is closely and negatively correlated with membranous E-cadherin expression according to the statistics in primary tumor lesions of HCC patients (Supplementary Table). As surgical treatment is not advocated once distant metastasis is detected in HCC patients, limited metastatic samples were obtained. In metastatic foci of eight independent HCC patients, the nuclear OPN was accompanied by membranous E-cadherin in five cases (Figure 6h). This phenomenon strongly suggested that nuclear OPN and epithelial status is closely related in metastases.

### VEGF in the microenvironment elicits OPN nuclear accumulation

**Figure 7:**
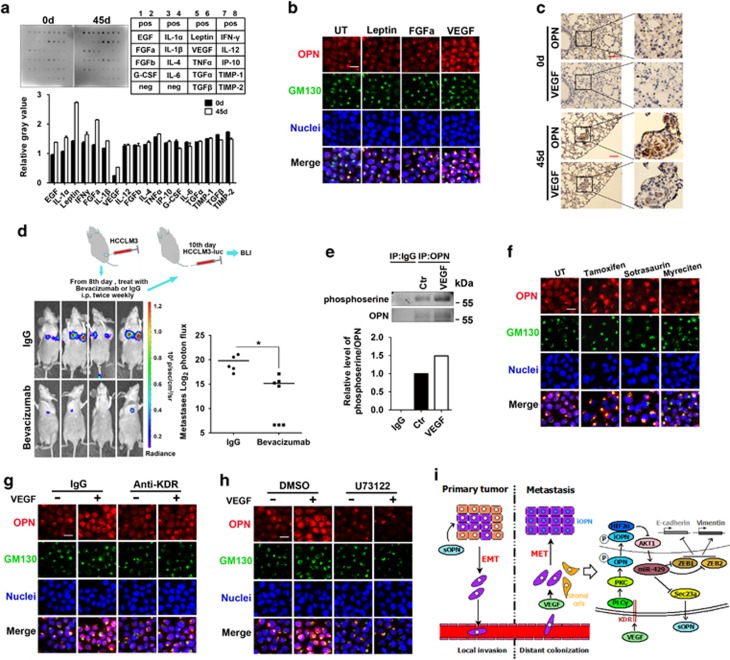
VEGF in the microenvironment elicits OPN nuclear accumulation. (**a**) Representative semi-qualitative assay of cytokines in homogenates from indicated lungs using mouse angiogenesis antibody array. Schematic diagram of the array is shown and spots were scanned for densitometry analysis using the Quantity One software (Bio-Rad, Hercules, CA, USA). Data represent mean±S.D. (**b**) Immunofluorescence staining of OPN and GM130 in HCCLM3 cells after treatment with Leptin (100 ng/ml), FGFa (50 ng/ml), or VEGF (200 ng/ml). Scale bar, 10 *μ*m. (**c**) Representative images of IHC staining in indicated metastases. Scale bars, 100 *μ*m. (**d**) Representative bioluminescent images and the log2 luminescent photon flux of mice, which were treated as the diagram. *P<0.05. (**e**) Immunoblot analysis of phosphoserine and OPN in HCCLM3 cells stimulated as indicated. The ratio of gray values of phosphoserine and OPN were compared. Ctr, control. (**f**, **g** and **h**) Immunofluorescence staining of OPN and GM130 in HCCLM3 cells. (**f**) Cells were treated with PKC inhibitors, Tamoxifen (6 *μ*M), Sotrasaurin (5 *μ*M), or Myreciten (10 *μ*M). (**g**) Cells were treated with the indicated antibody (300 ng/ml) and VEGF (200 ng/ml). (**h**) Cells were treated with the PLC*γ* inhibitor U73122 (20 *μ*M) and VEGF (200 ng/ml). Scale bars, 10 *μ*m. (**i**) The schematic model of sOPN and iOPN as facilitators of tumor metastasis by inducing EMT and MET separately during the invasion-colonization cascade

On the basis of the above observations, the mechanism by which OPN enters the nucleus in metastases aroused our interest. A receptive microenvironment at the destination site is pivotal for the engraftment of disseminated tumor cells. In response to some soluble factors, tumor-associated cells, such as haematopoietic progenitors, initiate the ‘premetastatic niche' by collaborating with other cell types, such as stromal cells. This establishes a premetastatic microenvironment containing cytokines and matrix-degrading enzymes.^25, 26, 27^ We subcutaneously injected HCCLM3 cells and harvested lung tissues on the 1st, 15th, 30th, and 45th day. The harvested lung tissues were homogenized and subjected to cytokine detection. Among all the cytokines detected, densitometry values of Leptin, FGFa, and VEGF increased obviously over time, especially between the 1st and 45th day (Figure 7a; Supplementary Figure S5). Dual immunofluorescence staining of HCCLM3 cells stimulated with Leptin, FGFa, or VEGF showed that neither Leptin nor FGFa enabled OPN to accumulate in nuclei, whereas interestingly, in cells stimulated by VEGF, obvious accumulation of OPN in nuclei was observed (Figure 7b). In addition, IHC staining showed that VEGF was distributed in the stoma surrounding tumor cells with the nuclear accumulation of OPN (Figure 7c). To further validate the impact of VEGF *in vivo*, an animal model was established as shown in Figure 7d, and bioluminescence imaging assay showed the injection of VEGF antibody impeded lung metastasis. These data highlighted the important role of VEGF in the induction of OPN nuclear accumulation and distant metastasis.

We next tried to explore the mechanism how OPN in metastatic tumor cells translocated to nuclei. Protein shuttling between the nucleus and cytoplasm influences cell function; and posttranslational modifications, especially phosphorylation and acetylation, critically regulate the subcellular localization of intracellular proteins.^28, 29^ We used prediction servers (http://www.cbs.dtu.dk/services/NetPhos and http://www.cbs.dtu.dk/services/NetAcet) to predict the modifications of OPN and found that only phosphorylation (notably phosphoserine) may occur. The immunoprecipitation assay was then performed with anti-OPN antibody followed by immunoblot analysis with anti-phosphoserine antibody. After HCCLM3 cells were stimulated by VEGF, OPN was of more phosphoserine (Figure 7e), suggesting that phosphorylation was associated with the nuclear translocation of OPN. Next the NetPhosK 1.0 server (http://www.cbs.dtu.dk/services/NetPhosK) was used to identify kinases that potentially induced phosphorylation of OPN. Integrating the PTM/Processing information of OPN obtained from the UniProt database, we identified PKC as the candidate. Thereafter, kinase inhibitor studies showed decreased nuclear distribution of OPN in cells treated with inhibitors of tamoxifen, sotrasaurin, or myricetin, which confirmed the involvement of PKC (Figure 7f).

The binding of VEGF to its receptors induces receptor dimerization and autophosphorylation, leading to the activation of several downstream molecules, including phospholipase C*γ* (PLC*γ*), protein kinase C (PKC), and phosphatidylinositol 3-kinase (PI3K).^30, 31^ Several studies have reported the VEGFR2 (KDR)/PLC*γ*/PKC pathway-mediated VEGF signaling.^32, 33^ We then performed antibody neutralization and kinase inhibitor studies, and immunofluorescence staining showed the treatment of KDR antibody or PLC*γ* inhibitor blocked the nuclear translocation of OPN (Figures 7g and h). All these findings suggested that VEGF induced the phosphorylation and nuclear import of OPN through the KDR/PLC*γ*/PKC-dependent pathway.

## Discussion

In this study, we investigated the expression of OPN in various cancer cells, and found not only sOPN but also iOPN. We then found that sOPN triggers EMT in primary sites, whereas iOPN induces MET in distant metastases. iOPN induces MET through the AKT1/miR-429/ZEB cascade following the combination with HIF2*α* in nuclei. We further found that VEGF is responsible for the nuclear accumulation of OPN in lung metastases through KDR/PLC*γ*/PKC-dependent pathway. Our findings support the distinct roles of secretory and iOPN in phenotypic plasticity during different stages of tumor metastasis: sOPN promoted EMT to initiate early metastatic dissemination, whereas iOPN evoked MET to facilitate metastatic colonization in the late stage of metastatic colonization.

In 1997, iOPN was initially found in rat calvarial cells by Sodek's group.^11^ They performed the single cell analysis and found two patterns of OPN staining: perinuclear and perimembrane distribution. The perinuclear OPN co-localizing with the Golgi apparatus was the typical sOPN, and iOPN showed perimembranous distribution. Later, the cytoplasmic and nuclear distribution of iOPN was demonstrated.^14, 15^ The intracellular immunostaining of OPN show four distinct patterns: perimembranous staining, nuclear retention, cytoplasm distribution, and perinuclear staining which represents sOPN. Cumulative research has revealed crucial roles of iOPN, including migration, cell cycle, and motility.^12, 13, 14^ However, research on the role of iOPN in cancer metastasis is still inconclusive.

Early in the metastatic cascade, cancer cells from the primary tumor undergo EMT, which endows non-invasive tumor cells with the ability to invade and disseminate.^1, 2^ In recent years, MET, the reversal of EMT, is believed to contribute to colonization at a secondary site. Versican was found to induce MET of breast cancer cells to favor metastatic development in the lungs.^34^ The potent miR-200s promoted MET by directly affecting the ZEB/E-cadherin axis, and high miR-200 expression was required for efficient colonization in secondary organs.^5, 18, 19, 35^ The overexpression of miR-429induced MET in metastatic ovarian cancer cells.^36^ Despite these findings, functional studies linking MET with metastatic colonization ability are relatively scarce.

AKT, also known as protein kinase B (PKB), is a central node in cell signaling downstream. The AKT family, including AKT1, AKT2, and AKT3, contributes to diverse cellular roles, such as cell survival, growth, proliferation, angiogenesis, and metabolism. AKT signaling has also been implicated in a variety of human cancers.^37^ Although three AKT isoforms are structurally homologous and share similar mechanisms of activation, they possess distinct features. AKT1 and AKT2 are extensively expressed, whereas AKT3 has a much more limited distribution. AKT1 and AKT2 have distinct roles in regulating EMT, and the ablation of AKT1 was correlated with the decreased abundance of miR-200s and the subsequent increase of ZEB and suppression of E-cadherin.^22, 23^ OPN usually activates AKT by the phosphorylation via kinase like ILK and PI3K.^20, 21^ In our study, nuclear OPN was found to regulate the level of AKT1 expression rather than the phosphorylation of AKT1. We demonstrated the negative regulation of HIF2*α* on AKT1 by ChIP assays and the combination of OPN and HIF2*α* by Co-IP assays. In conclusion, nuclear OPN can interact with HIF2*α* and impact the subsequent AKT1/miR-429/ZEB cascade.

The ‘seed and soil' hypothesis put forward by Steven Paget is a milestone in the study of cancer metastasis. It presents the notion that a conducive microenvironment is essential for malignant cells to engraft distant tissues and form metastases.^25, 26^ The ideas that specific organs are predisposed to metastases in certain cancers and that signaling between cytokines, chemokines, and their receptors regulates tumor cell homing to secondary organs are now well established.^27^ Recent research has demonstrated that the primary tumor itself is able to improve the microenvironment of secondary organs, termed ‘pre-metastatic niches', before the arrival of tumor cells.^25, 26, 27, 38^ The pre-metastatic niche was first described by Kaplan *et al.*^38^ In response to tumor-derived VEGF and PlGF, bone marrow-derived haematopoietic progenitor cells (HPCs) expressing VEGFR1 home to tumor-specific pre-metastatic sites and form cellular clusters in secondary organs. Clusters of VEGFR1^+^ HPCs expressing VLA-4, the fibronectin receptor, interact with resident fibroblasts to stimulate fibronectin production and secrete MMP9 to create fitting niches for the recruitment of disseminating CXCR4^+^ tumor cells. Subsequent studies have identified other factors and bone marrow-derived cells important in the formation of pre-metastatic niche.^25^ In our study, we demonstrated that VEGF could induce the nuclear accumulation of OPN via the KDR/PLC*γ*/PKC pathway, thus inducing MET and metastasis formation.

Canonical sOPN is widely recognized to be strongly associated with tumor metastasis, but the role of iOPN has not been fully elucidated. Therefore, a better understanding of the different types of OPN functioning in regulating tumor metastasis is of great importance to craft a more efficient treatment strategy.

## Materials and methods

### Cell culture

Human liver cancer cells (Hep3B and HepG2), human lung cancer cells (A549, SK-MES-1, NCI-H460, and NCI-H1299), and the transformed embryonic kidney cell line 293T were purchased from American Tissue Culture Collection (ATCC, Manassas, VA, USA). HCCLM3 and MHCC97-L were obtained from the Liver Cancer Institute, Zhongshan Hospital, Fudan University (Shanghai, China). Further detail was described in Supplementary Materials and Methods.

### Constructs

Lentiviral particles of short hairpin RNA (shRNA) were used to knockdown OPN. Lentiviral vectors encoding the intracellular form of human OPN (iOPN) were generated by using pLenti6/V5-TOPO system (Invitrogen, Carlsbad, CA, USA) and were designated as LV-iOPN. The iOPN cDNA was generated from full-length OPN cDNA by deleting the codons from 1 to 15. The empty vector was used as a negative control and was designated as LV-Non. Further details about shRNA and siRNA sequences, plasmids and cell infection are available in Supplementary Materials and Methods.

### ELISA

Cells were grown to 90% confluence, and culture media was harvested and subjected to the ELISA kit (QuantiKine assay; R&D, Minneapolis, MN, USA) to detect OPN concentration.

### Quantitative real-time PCR

For mRNA analysis, total RNA was isolated using EASYspin Plus tissue/cell RNA extraction kit (RN28; Aidlab, Beijing, China). First-strand synthesis was performed with the PrimeScript RT reagent kit (RR037A; Takara, Dalian, China). And qPCR was performed using SYBR Green reagent (ABI, Paisley, UK) in the ABI 7500 qPCR System. Reactions were performed twice in triplicate, and actin values were used to normalize gene expression. For miRNA analysis, total RNA was extracted using Trizol reagent (Invitrogen). Mature miRNAs were reverse transcribed (ABI), and qPCR was performed using TaqMan miRNA assays (ABI). U6 values were used to normalize miRNA expression. The list of primers used is provided in Supplementary Table S3.

### Immunoblotting, Co-IP analysis, and immunofluorescence staining

Immunoblotting, Co-IP analysis, and immunofluorescence staining were performed as described in Supplementary Materials and Methods. The primary and secondary antibodies are listed in Supplementary Table S4.

### Luciferase reporter assays

Luciferase activity was measured as described in Supplementary Materials and Methods.

### ChIP assay

Four days after Hep3B cells were transfected with the indicated lentivirus, about 3 × 10^6^ cells were harvested and subjected to the ChIP assay according to the manufacturer's instructions (Millipore, Billerica, MA, USA). The samples were incubated with the anti-HIF2*α* antibody (6 *μ*g; Abcam, Cambridge, MA, USA) overnight at 4 °C. PCR was performed for 35 cycles with the annealing temperature at 55 °C. The amplification products were analyzed by electrophoresis with 1.5% agarose gels. The primers used are listed in the Supplementary Table S5.

### Animal studies

Animal care and experimental procedures were approved by Shanghai Medical Experimental Animal Care Commission. Male athymic BALB/c nude mice (4–6 weeks old) were purchased from Shanghai Experimental Animal Center of Chinese Academic of Sciences and maintained in specific pathogen-free conditions. Detailed experimental procedures were described in Supplementary Materials and Methods.

### HCC samples, histology and IHC

Human HCC primary tumor slides were obtained from Guangxi Cancer Hospital (Nanning, Guangxi, China). Human HCC metastatic tumor slides were provided by Shanghai Xinchao Biotechnology (Shanghai, China). Informed consents were obtained, and the procedure was approved by the hospital ethical committee. Hematoxylin and eosin and IHC were carried out routinely and the detailed protocol was described in Supplementary Materials and Methods.

### Plate colony-forming assay and cytokine detection assay

Plate colony-forming assay and cytokine detection assay were performed as described in Supplementary Materials and Methods.

### Statistical analysis

Results are presented as mean±S.D. Quantitative variables were analyzed by the two-tailed Student's *t*-test and the Wilcoxon-rank sum test. Statistical significance was set at two-sided *P*<0.05.
