# Supplementary material for: Osteopontin facilitates tumor metastasis by regulating epithelial–mesenchymal plasticity
Source: Cell Death Dis. 2016 Dec 29;7(12):e2564–. doi: 10.1038/cddis.2016.422 (PMC5261026; doi:10.1038/cddis.2016.422)
Supplement: Supplementary Information [file cddis2016422x1.docx]

**Supplementary Information**

**Supplementary Materials and Methods**

**Cell culture**

Human colorectal cancer cells (Caco-2, LS 174T, LoVo, and COLO 205), human breast cancer cells (MCF7, SK-BR-3, BT-20, and MDA-MB-231), human liver cancer cells (Hep3B and HepG2), human lung cancer cells (A549, SK-MES-1, NCI-H460, and NCI-H1299), and 293T cells were purchased from American Tissue Culture Collection (ATCC, Manassas, VA, USA). HCCLM3 and MHCC97-L were obtained from the Liver Cancer Institute, Zhongshan Hospital, Fudan University (Shanghai, China). NCI-H460, NCI-H1299, and COLO 205 were cultured in RPMI-1640 medium (Gibco, Grand Island, NY, USA) supplemented with 10% fetal bovine serum (Gibco) at 37 °C in a humidified incubator containing 5% CO2. Caco-2 was cultured in Dulbecco’s modified Eagle’s medium (DMEM, Gibco) supplemented with 20% fetal bovine serum (Gibco) at 37 °C in a humidified incubator containing 5% CO2. Other cell lines were cultured in DMEM (Gibco) supplemented with 10% fetal bovine serum (Gibco) at 37 °C in a humidified incubator containing 5% CO2.

**Constructs**

Lentiviral particles of short hairpin RNA (shRNA) were used to knock down OPN. We generated plasmid vectors encoding short hairpin RNAs (shRNAs) targeting OPN, or scramble shRNA using pENTR/U6 (Invitrogen, Carlsbad, CA, USA), and designated them as pshOPN and pshNon, respectively. We generated lentiviral vectors encoding shRNAs using pLenti6/BLOCKiT-DEST (Invitrogen) by the Gateway cloning system and ViraPower Packaging Mix (Invitrogen), and designated them as lentiviral vector shOPN and shNon, respectively. The sequences targeting human OPN for RNA interference were as follows (5’**→**3’): shOPN-1 (top strand, ccggGGCTGATTCTGGAAGTTCTGAtcaagagTCAGAACTTCCAGAATCAGCCtttttg, and bottom strand, gatccaaaaaGGCTGATTCTGGAAGTTCTGActcttgaTCAGAACTTCCAGAATCAGCC), shOPN-2 (top strand, ccggGTGGGAAGGACAGTTATGAAAtcaagagTTTCATAACTGTCCTTCCCACtttttg, and bottom strand, gatccaaaaaGTGGGAAGGACAGTTATGAAActcttgaTTTCATAACTGTCCTTCCCAC), (Capital letters are target sequences of OPN). For negative control, we used sramble shRNA (top strand, ccggAATTCTCCGAACGTGTCACGTtcaagagACGTGACACGTTCGGAGAATTtttttg, and bottom strand, gatccaaaaaAATTCTCCGAACGTGTCACGTctcttgaACGTGACACGTTCGGAGAATT).

Commercially synthesized small interfering RNA targeting human AKT1, AKT2, HIF2α, and negative control siRNA (siAKT1, siAKT2, siHIF2α, and siNC) was generated by GenePharma (Shanghai, China), in addition to miR-429 and miR-141 inhibitors and mimics. The sequences of the above are listed in the Supplementary Table S1 and S2.

HA-HIF2alpha-pcDNA3, purchased from Addgene (#18950), contained HA-tag on the N-terminal end and was designated as HA-HIF2α. The expression plasmid for iOPN was generated by inserting iOPN cDNA into the pcDNA3.1/V5-His B vector (Invitrogen) and was designated as V5-iOPN. Transfections were performed using X-tremeGENE^®^ HP DNA transfection reagent (Roche) following the manufacturer’s instructions.

**Immunoblotting (Western Blot analysis)**

Cells were lysed in 1× SDS lysis buffer (62.5 mM Tris-HCl, 2% w/v SDS, 10% glycerol, 50 mM DTT, 0.01% w/v bromophenol blue), sonicated and subsequently boiled for 8 minutes. Proteins at the equal amount were separated by 10% SDS-PAGE and transferred onto polyvinylidenedifluoride (PVDF) membranes (0.45 μm). The membrane was blocked with 5% fat-free milk for 1 hour at room temperature, and probed with primary antibody at 4 °C overnight. Rinsed 3 times with TBST buffer, the membrane was incubated with the corresponding HRP-conjugated secondary antibody for 1 hour at room temperature. Finally Immobilon Western Chemiluminescent HRP Substrate (Millipore, Billerica, MA, USA) were used for detection. Prepare the chemiluminescence reagent (0.1 ml of reagent per cm^2^ of membrane) by mixing equal volumes of Luminol Reagent and Peroxide Solution and add the substrate onto the blot. Incubate the blot for 2 minutes at room temperature with protection from light. The chemiluminescent signals on the blot were detected by the ChemiDoc™ Imaging Systems (Bio-Rad, Hercules, California, USA). If necessary, fresh HRP substrate can be added to the same blot for consecutive exposures. The antibodies used were addressed in the Supplementary Table S4.

**Co-IP analysis**

Cells of about 3 × 10^6^ were harvested in cell lysis buffer for Western and IP (P0013; Beyotime) supplemented with cOmplete Protease Inhibitor Cocktail (Roche), Phosphatase Inhibitor Cocktail (Biotool), and PMSF (Beyotime). The cell lysate was centrifuged at 12,000 rpm at 4°C for 15 minutes and the supernatant were incubated with protein A/G agarose beads (Santa Cruz) and normal IgG as a pretreatment. Precleared lysates were then incubated with anti-V5, anti-HA, anti-OPN, or anti- HIF2α antibodies (4 μg) for 1 hour, then incubated overnight with protein A/G agarose beads. The beads were collected by centrifugation, washed six times with the lysis buffer and resuspended in 3×SDS loading buffer. The immunoprecipitates were eluted from the beads by incubation at 95°C for 5 min. The eluted proteins were separated by SDS-PAGE and Western blotting was subsequently performed with the corresponding primary and secondary antibodies. Details about antibodies used are described in the Supplementary Table S4.

**Conditioned media (CM) preparation**

HCCLM3 cells were cultured in DMEM medium (Gibco) containing 10% FBS. 24 hours later, medium was removed and fresh medium was added. Another 24 hours later, condition medium was harvested and clarified by centrifugation.

**Immunofluorescence staining**

For confocal studies, cells were grown on glass chamber slides; for inverted fluorescence microscopy, cells were plated in 24-well cell culture plates. After the indicated treatments, cells were washed with PBS and fixed with 10% buffered formalin for 15 minutes. Cells were then incubated with blocking buffer (1.25 ml of FBS, 23.75 ml of PBS, 75 μl of Triton-100, and ddH_2_O in a total volume of 50 ml) for 30 minutes. Then, the primary antibody was incubated with cells at 4 °C overnight. The next day, cells were rinsed with PBS and incubated with the appropriate fluorescence-conjugated secondary antibody at room temperature for 1 hour. DAPI (4, 6-diamidino-2-phenylindole, Dojindo Laboratories) was used to manifest nuclei. Images were captured using confocal microscopy TCS SP5 (Leica Microsystems) or a Leica DMIRB fluorescence microscope (OLYMPUS IX71).

**Luciferase reporter assays**

Cells were transfected with pGL3 reporter plasmid and full or various truncated AKT1 promoter-luciferase constructs with the use of X-tremeGENE HP Transfection Reagent (Roche) according to the manufacturer’s instructions. The pRL-TK plasmid (encoding Renilla luciferase) was cotransfected as a control for transfection efficiency. Cells were harvested 48 hours later, and luciferase activity was measured using the Dual Luciferase Reporter Assay System (Promega).

**Animal studies**

Animal care and experimental procedures were approved by the Shanghai Medical Experimental Animal Care Commission. Male athymic BALB/c nude mice (4-6 weeks old) were purchased from the Shanghai Experimental Animal Center of Chinese Academic of Sciences and maintained in specific pathogen-free conditions. In the first model, HCCLM3 cells (5 × 10^6^) infected with shRNA or without infection were implanted subcutaneously (9/group). In the second model, HCCLM3 cells labeled with luciferase (2 × 10^6^) infected with shRNA were injected via the tail vein. In the third model, HCCLM3 cells (5 × 10^6^) were injected subcutaneously into four groups (the 1st day, the 15th day, the 30th day, and the 45th day group). Mice were sacrificed and lung tissues were prepared for cytokine detection or tissue sectioning. In the fourth model, HCCLM3 cells (5 × 10^6^) were injected subcutaneously, and from the 8th day on, mice were treated with Bevacizumab or control IgG at 10 mg/kg by intraperitoneal injection twice weekly. On the 10th day, HCCLM3 cells labeled with luciferase (2 × 10^6^) were injected intravenously. Lung metastases were manifested on the 40th day by bioluminescence imaging by IVIS 100 Imaging System (Xenogen) after intraperitoneal injection of D-Luciferin Potassium salt (SYNCHEM). All the mice were sacrificed, and lungs and primary tumors were collected to make consecutive sections. In the first model, primary cells from both primary tumor lesions and lung metastatic colonies were harvested, cultured, and subsequently detected.

**Histology and immunohistochemistry (IHC)**

Primary tumor foci or lungs were excised, fixed in 10% neutral-buffered formalin for 24 hours, embedded in paraffin, sectioned at 2-µm thickness and stained with hematoxylin and eosin (H&E). For IHC, the slides were dewaxed, hydrated and washed, and the endogenous peroxidase activity was quenched after treatment with 0.3% hydrogen peroxide for 20 minutes at room temperature. After microwave antigen retrieval, slides were blocked and then incubated with the primary antibody at 4 °C overnight. Subsequently, sections were rinsed and incubated with the working solution of horseradish peroxidase-labeled secondary antibody for 60 minutes at room temperature. After three rinses, staining was visualized using the peroxide substrate solution 3, 3’-Diaminobenzidine (DAB). Counterstained by hematoxylin, the slides were dehydrated in graded alcohol and mounted. Negative controls were prepared in the absence of primary antibody. Evaluation of immunostaining was independently conducted by two pathologists.

**Plate colony-forming assay**

Primary cells were isolated and cultured by trypsin digestion from mouse lungs in the first animal model. Following 3 passages, HCCLM3 cells infected with shOPN and shNon were dispersed in a single-cell suspension, which was prepared and inoculated in 10-cm dishes with a density of 5 × 10^3^ cells and maintained for 30 days. Then, the colonies were stained with 0.1% crystal violet for 20 minutes after fixation with 4% paraformaldehyde for 5 minutes, and the colonies were dried and photographed.

**Cytokine detection assay**

After sacrifice, the left lung lobes were perfused with 10% neutral-buffered formalin, placed in fixative for approximately 24 hours, then paraffin-embedded and sectioned serially. The right lung lobes were snap-frozen (in liquid nitrogen) and stored at -80°C until further analysis. The frozen lungs were thawed and transferred to chilled T-PER (Thermo Fisher) containing cOmplete Protease Inhibitor Cocktail (Roche) at a proportion of 1ml T-PER per 0.1 g tissue. The lung tissues were homogenized and then centrifuged at 9,000 × g for 10minutes at 4 °C. Supernatants were transferred to frozen Eppendorf tubes. Total protein concentrations in the homogenates were determined using a BCA kit (Beyotime). Samples containing 4 mg total proteins were subjected to the Mouse Angiogenesis Antibody Array (Affymetrix, Panomics) to assess the relative levels of cytokines. All the procedures were conducted according to the manufacturer’s instructions. Chemiluminescence is detected in the same manner as Western blot analysis.

**Mass spectrometry (MS)**

HCCLM3 cells transfected with siNC, siSec23a-1, and siSec23a-2 were cultured in DMEM medium (Gibco) containing 10% FBS. 24 hours later, medium was removed and fresh complete medium was added. Following one population doubling, cells were cultured in DMEM medium without FBS for 48 hours and then medium was harvested and clarified by centrifugation for further analysis. The collected media were first ultrafiltrated using Amicon^®^ Ultra-15 centrifugal filter devices at 3, 500 rpm for 1 hour in 4 °C atmosphere. Afterwards, the concentrated samples were subjected to protein denaturation solution (8 M Guanidine hydrochloride, 5 mM EDTA, 0.5 M Tris) and 1 M DTT for 1 hour at 60 °C. Following that, samples were treated with 2.9 M Sodium iodoacetate for 45 minutes at room temperature for reduction and alkylation of disulfide bonds. Then, samples were desalted and buffer-exchanged with weak-alkaline NH_4_CO_3_ solution by AKTA AVANT 25 system. The eluted fractions were monitored and collected through UV detectors at wavelength of 280/214 nm, and then were digested with Trypsin (10 μg/ml) for 2 hours at 37 °C and the concentrations of the proteins were determined by the BCA method. Then samples with equal amounts of total proteins were dried under vacuum before methylation isotope labeling of carboxyl terminus. Deuterated methanol was used to label the Sec23a-silenced samples whereas natural methanol was used to label the control. The lyophilized peptides were incubated with freshly prepared reagents for 2 hours at room temperature. The sample was dried and the methyl ester reaction process was repeated twice. The reactions were terminated by vacuum freeze-drying. Finally, the deuterated methylated and methylated peptides were mixed after equivoluminally dissolving with 0.1% formic acid (FA) solution separately. The labeled peptides were centrifuged at 13, 300 rpm to remove possible impurities and subsequently identified and MS detected by Xevo G2-S QTOF analysis system. Each experiment was carried out three times to avoid experimental error in the quantitative process. The collected data were processed and target peptides with high specificity were identified and quantitated based on the UniProt knowledgebase.

**Supplementary Tables**

**Table S1.** Sequence of siRNA.

| **siRNA Sense(5’→3’) Antisense(5’→3’)** |
| --- |
| siNC UUCUCCGAACGUGUCACGUTT ACGUGACACGUUCGGAGAATT  siAKT1-1 GCACUUUCGCAAGGUGAUTT AUCACCUUGCCGAAAGUGCTT  siAKT1-2 AGGAAGUCAUCGUGGCCAATT UUGGCCACGAUGACUUCCUTT  siAKT2-1 GCGGAAGGAAGUCAUCAUUTT AAUGAUGACUUCCUUCGCTT  siAKT2-2 GCUCCUUCAUUGGGUACAATT UUGUACCCAAUGAAGGAGCTT  siHIF2α-1 GGAGCUAACAGGACAUAGUTT ACUAUGUCCUGUUAGCUCCTT  siHIF2α-2 CGCUCAGCCUAUGAAUUCUTT AGAAUUCAUAGGCUGAGCGTT  siSec23a-1 GGGUGAUUCUUUCAAUACUTT AGUAUUGAAAGAAUCACCCTT  siSec23a-2 GGUUGUCAAUCAGCAUAAUTT AUUAUGCUGAUUGACAACCTT |

**Table S2.** Sequences of miRNA mimics, miRNA inhibitor, and NC (negative control).

| **miRNA Sense(5’→3’) Antisense(5’→3’)** |
| --- |
| miR-429 UAAUACUGUCUGGUAAAACCGU GGUUUUACAGACAGUAUUAUU mimics  miR-141 UAACACUGUCUGGUAAAGAUGG AUCUUUACCAGACAGUGUUAUU mimics  miR-429 ACGGUUUUACCAGACAGUAUUA  inhibitor  miR-141 CCAUCUUUACCAGACAGUGUUA  inhibitor  NC CAGUACUUUUGUGUAGUACAA |

**Table S3.** Primers for qPCR.

| **Primer Forward (5’→3’) Reverse (5’→3’)** |
| --- |
| β-actin CGTGGACATCCGTAAAGACC ACATCTGCTGGAAGGTGGAC  OPN CTCCATTGACTCGAACGACTC CAGGTCTGCGAAACTTCTTAGAT  ZEB1 CTACAACAACAAGACACTGCTGT TGTTCTTTCAGAGAGGTAAAGCG  ZEB2 CAAGAGGCGCAAACAAGCC GGTTGGCAATACCGTCATCC  AKT1 ATGAGCGACGTGGCTATTGTGAAG GAGGCCGTCAGCCACAGTCTGGATG  HIF2α TGGCCGCTCAGCCTATGAAT TGGGTCTCCAGCCACACGTA  Sec23a GGACTGCTGGAGTGTACTTTTCCCA GCAGCTCGATTAGCCAATGCTTCA |

**Table S4.** Antibodies

| **Protein Cat. and company Usage Dilution** |
| --- |
| E-cadherin 3195, Cell signaling technology WB 1:1000  IF/IHC 1:100  Vimentin 3932, Cell signaling technology WB 1:1000  IF 1:100  N-cadherin sc-7939, Santa Cruz Biotechnology WB 1:1000  α-SMA A5228, Sigma-Aldrich WB 1:1000  ZEB1 3396, Cell signaling technology WB 1:1000  ZEB2 sc-48789, Santa Cruz Biotechnology WB 1:1000  OPN AF1433, R&D Systems WB 1:1000  IF/IHC 1:100  OPN sc-10591, Santa Cruz Biotechnology IP 1:40  GAPDH KC5G5, KangChen Bio-tech WB 1:2000  AKT1 2938, Cell signaling technology WB 1:1000  AKT2 3063, Cell signaling technology WB 1:1000  p-AKT1 05-669, Merck-Millipore WB 1:1000  HIF2α 7096, Cell signaling technology WB 1:1000  HIF2α ab199, Abcam IP/ IF 1:100  ChIP 1:100  HA H3663, Sigma-Aldrich WB 1:1000  IP 1:100  V5 V8137, Sigma-Aldrich WB 1:1000  IP 1:100  VEGF AF-493-NA, R&D Systems IHC 1:40  phosphoserine 05-1000X, Millipore WB 1:500 |

**Note:**

Secondary antibodies used in Western blot analysis, goat anti-rabbit IgG-HRP (sc-2030), goat anti-mouse IgG-HRP (sc-2031), and donkey anti-goat IgG-HRP (sc-2020) were purchased from Santa Cruz Biotechnology.

Secondary antibodies used in Co-IP analysis, Mouse TrueBlot^®^ ULTRA: Anti-Mouse Ig HRP (18-8817-33) and Rabbit TrueBlot^®^: Anti-Rabbit IgG HRP (18-8816-33) were purchased from Rockland Immunochemicals, Inc.

Secondary antibodies used in immunofluorescence, Alexa Fluor 555 donkey anti-goat IgG, Alexa Fluor 488 donkey anti-rabbit IgG, and Alexa Fluor 555 donkey anti-rabbit IgG were purchased from Life technologies.

Abbreviations: WB, Western blot analysis; IF, immunofluorescence; IHC, immunohistochemistry; Co-IP, co-immunoprecipitation.

**Table S5.** Primers used in ChIP assay.

| **Primer Forward (5’→3’) Reverse (5’→3’)** |
| --- |
| Fragment 1 TACAGACGGGGAAACTGAGGC GGAGCCGCCCGCCCACATCCGC  Fragment 2 AGAGAAGGGACGCCGCCGGGCCT TCCTTTGTGGCTAGCCTGGGTAC  Fragment 3 GTGTATACGTTTCTGTGCAGACGT GCAGGGCTCCGAGCCGCGGAC |

**Supplementary Figure Legends**

Figure S1. Expression of OPN in colon cancer and breast cancer cells.

(**a**) Immunofluorescence staining of OPN and nuclei in colon cancer cells (upper) and breast cancer cells (lower). Scale bars, 10 µm. (**b**) The cell culture media of cancer cells were collected and the level of secreted OPN was quantitated by ELISA. OPN concentrations of group Ӏ, ӀӀ, and ӀӀӀ cells are also displayed. (**c**) Fold-change in mRNA level of OPN in indicated cells. Samples were measured in duplicate and data represent mean ± S.D.

Figure S2. Alterations of transcription factors that regulate the epithelial-mesenchymal plasticity.

(**a, b, and c**) Relative mRNA expression levels of the transcription factors in type Ӏ cells (**a**), type ӀӀӀ and ӀӀ cells (**b and c**) which were transfected with the denoted lentivirus. Data represent mean ± S.D. *, *P* < 0.05, **, *P* < 0.01.

Figure S3. miR-141 does not play a core role in MET induced by iOPN.

(**a**) Immunoblot analysis of E-cadherin and Vimentin in Hep3B cells simultaneously transfected with indicated lentivirus and miR-429 mimics or inhibitor. (**b**) Fold-changes in mRNA levels of ZEB1 and ZEB2 in Hep3B cells treated as represented. Data represent mean ± S.D. (**C**) Immunofluorescence staining for E-cadherin and Vimentin in Hep3B cells treated as indicated. Scale bar, 10 μm.

Figure S4. miR-429 overexpression suppresses Sec23a-mediated secretion of OPN.

(**a and b**) Relative expression of miR-429 in the primary tumor and metastases (**a**), and in original HCCLM3 and the lung-metastatic HCCLM3 (HCCLM3-lung) cells (**b**). Tissues and cells were acquired from mice that were subcutaneously injected with HCCLM3 cells. Data represent mean ± S.D. (**c**) Relative mRNA/miRNA expression of HCCLM3 cells transfected with NC or miR-429 mimics. Data represent mean ± S.D. (**d**) Mass spectrometry analysis of the abundance of secreted OPN in the conditioned media from HCCLM3 cells with Sec23a knockdown. Red arrows mark secreted OPN and values are displayed. (**e**) Immunoblot analysis of secreted OPN in the conditioned media from indicated cells. (**f**) Immunofluorescence staining of OPN in indicated cells. Scale bar, 10 µm. (**g**) Fold -change in mRNA level of Sec23a and OPN in indicated cells. Data represent mean ± S.D.

Figure S5. Cytokine detection assay of homogenates from lungs.

Cytokines in homogenates from lungs acquired on the indicated time points were assayed using the Mouse Angiogenesis Antibody Array. Schematic diagram is shown and the spots in membranes were scanned and quantitated for densitometry analysis. Data represent mean ± S.D.
