# Supplementary figures and images for: Osteopontin facilitates tumor metastasis by regulating epithelial–mesenchymal plasticity
Source: Cell Death Dis. 2016 Dec 29;7(12):e2564–. doi: 10.1038/cddis.2016.422 (PMC5261026; doi:10.1038/cddis.2016.422)

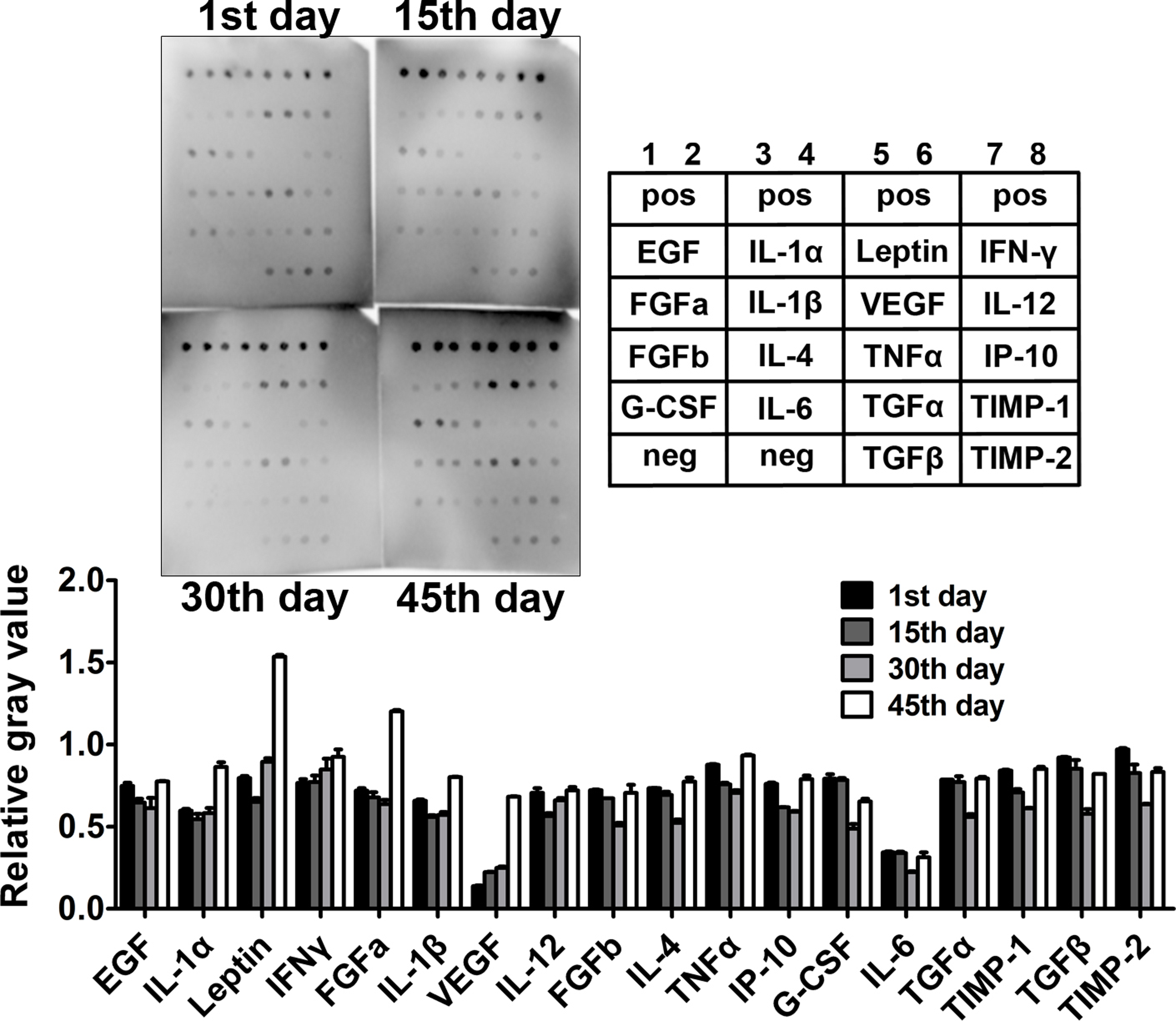

Supplement: Supplementary Figure S5 [file cddis2016422x6.tif]

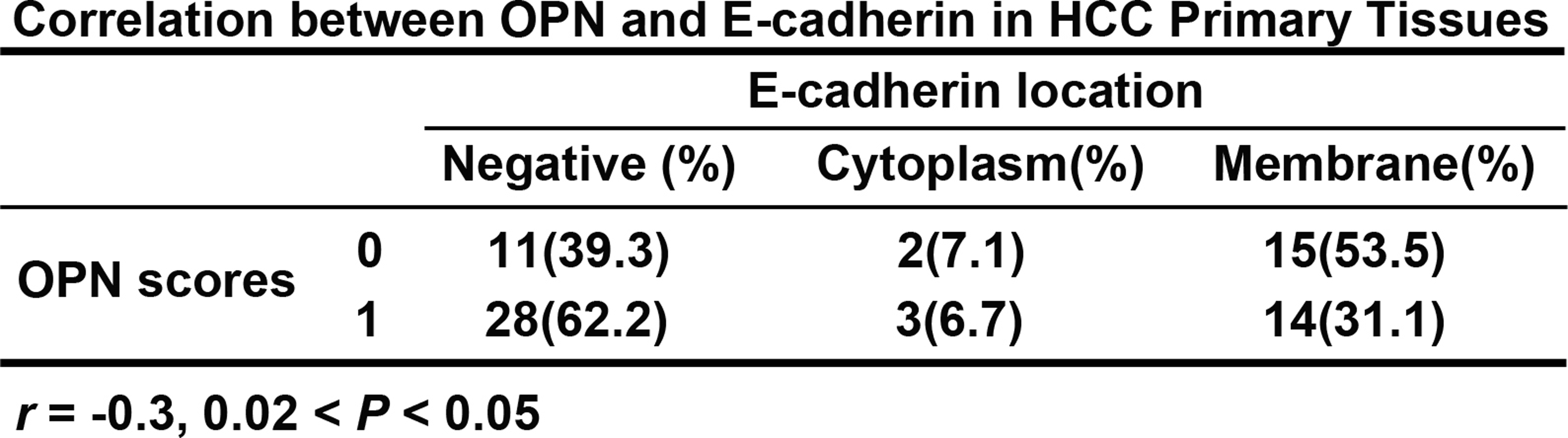

Supplement: Supplementary Table [file cddis2016422x7.tif]
